# A 26-Year-Old Man with Sternoclavicular Arthritis

**DOI:** 10.1371/journal.pmed.0030293

**Published:** 2006-08-29

**Authors:** Christian Gutierrez Ruiz, J. Jaime Miranda, Georgios Pappas

## Abstract

A 26-year-old man presented to a hospital in Lima, Peru, with a two-week history of fever, myalgias, and arthralgia of the left hip and right sternoclavicular joint. The authors discuss the work up, diagnosis, and management.

## DESCRIPTION of CASE

A 26-year-old man presented to the Hospital San Juan de Matucana, Lima, Peru, with a two-week history of fever, myalgias, and arthralgia of the left hip and right sternoclavicular joint. On clinical examination, localized tenderness over the sacroiliac joint and reproduction of Gaenslen's sign (lumbosacral tenderness on hip hyperextension with contralateral hip flexion) and Patrick's sign (localized pain on flexion, abduction, and external rotation of ipsilateral hip) were suggestive of sacroiliitis. The sternoclavicular joint was tender on palpation, but there was no overlying erythema or edema.

Four months prior to presentation, the patient had been diagnosed with brucellosis following the consumption of non-pasteurized goat cheese in Huarochiri, an area within the Sierra of Lima that is hyperendemic for brucellosis. At presentation to the Hospital San Juan de Matucana, no data were available on the accuracy of the diagnostic methods used to make a diagnosis of brucellosis. The patient did not recall any joint symptoms when he had first been diagnosed with brucellosis. He reported that he was treated with oral rifampin 900 mg and doxycycline 200 mg daily for five weeks, with rapid resolution of his symptoms.

### What Was the Differential Diagnosis of the Joint Symptoms?

A young patient presenting with a febrile syndrome associated with arthralgias and myalgias could suffer from a wide variety of inflammatory processes, including inflammation due to various viral and bacterial infections. The localization of the patient's arthralgia in the hip and supraclavicular joints suggests that the arthralgias did not belong to simply constitutional symptomatology, but were more likely to be due to arthritis (i.e., joint inflammation). The differential diagnosis of acute and chronic arthritis is shown in Box 1.

**Box 1. Differential Diagnosis of Acute and Chronic Arthritis**

**Acute Arthritis**

Septic

- *Neisseria gonorrhoeae*
- *Staphylococcus aureus*
- *Streptococcus* spp. (especially *S. pyogenes*)
- *Haemophilus influenzae*
- Gram-negative bacteria (*Pseudomonas aeruginosa*, *Escherichia coli*, *Salmonella typhi*)
- *Brucella* spp.
- Lyme disease

Inflammatory

- Gout and pseudogout (in elderly or predisposed individuals)
- Autoimmune disorders in the presenting stage (rheumatoid arthritis, systemic lupus erythematosus)
- Seronegative arthritis (psoriatic, related to inflammatory bowel disease, Still disease)
- Reactive arthritis
- Rheumatic fever

Traumatic (including hemarthrosis)

**Chronic Arthritis**

- Infectious (including chronic gonococcal arthritis, *Brucella*, syphilis, tuberculosis, Lyme disease, and fungal arthritis)
- Gout and pseudogout
- Autoimmune disorders (rheumatoid arthritis, seronegative arthritis)
- Osteoarthritis

#### Septic arthritis

This patient's relatively protracted fever and oligoarthritis could be the presenting symptoms of a bacterial infection [1]. Neisseria gonorrhoeae is an obvious possible diagnosis, although one would expect a different distribution of the affected joints [2], most commonly affecting wrists, ankles, and elbows, and there would usually be a more severe clinical presentation after a two-week history of disease.

A staphylococcal infection, with arthritis secondary to bacteremia-related seeding, is another diagnostic possibility, but there was no relevant history of trauma or skin and soft-tissue infections. In addition, after a two-week history of the disease, one would expect a more severe clinical presentation. The same is true for streptococcal infections. Nevertheless, staphylococcal arthritis may sometimes follow an indolent course, and an entry point or event may not be present in the history, and so staphylococcal arthritis was a possibility in this patient.

Typhoid is endemic to many parts of Peru, and so was another diagnostic possibility. The possibility of Lyme disease would have been higher in a different geographical setting. Mycobacterial and fungal arthritis may present in a chronic form, but mainly in immunocompromised patients. No predisposing factors to endocarditis were present.

#### Autoimmune and seronegative arthritis

The constellation of this patient's symptoms might represent the first symptoms of systemic lupus erythematosus, although other prominent disease characteristics would have to be present to make the diagnosis. The possibility of rheumatoid arthritis should also be entertained, as should other causes of a seronegative arthritis, although a diagnosis of psoriatic arthritis would require relevant dermatological findings, and a diagnosis of adult-onset Still disease usually demands exclusion of other underlying inflammatory processes. Peripheral oligoarthritis can occur in the course of inflammatory bowel disease; furthermore, sacroiliitis is a common extra-intestinal manifestation of inflammatory bowel disease. This patient, however, had no history of gastrointestinal symptoms.

#### Reactive arthritis

This patient was in the right age range for a diagnosis of reactive arthritis, but he did not have obvious symptoms of a preceding gastrointestinal or genitourinary infection four to six weeks prior to his joint symptoms. Nevertheless, he might have had a mild, subclinical preceding gastrointestinal or genitourinary infection that triggered reactive arthritis.

#### Other possible causes for the patient's joint symptoms

Other causes of arthritis, such as gout and pseudogout, were very unlikely given the patient's young age. Brucellosis remained a possible cause for his symptoms. The patient was initially treated with an optimal regimen for treating brucellosis, but for a sub-optimal period, and the possibility of brucellosis relapse should be prominent in the list of provisional diagnoses, even if protracted fever was the only presenting symptom. The diagnostic possibility of brucellosis is further strengthened by the presence of sacroiliitis, which is a common complication of brucellosis.

### What Tests Are Now Indicated in This Patient?

Imaging studies should be performed to confirm the clinical diagnosis and determine the severity of the sacroiliitis, while sternoclavicular involvement should also be investigated radiologically. Plain radiographs can often be normal in arthritis of both these sites, and, if indicated, more sophisticated techniques, such as magnetic resonance imaging of the sacroiliac joint and ultrasound of the sternoclavicular joint, should be employed.

If arthritis of the sternoclavicular joint was radiologically confirmed, attempts should be made to aspirate joint fluid for cultures, white cell count, and biochemistry tests. Interpretation of these tests is shown in Table 1. For any patient in whom there is a clinical suspicion of septic arthritis, all attempts should be made to obtain samples for culture, and the relevant serological studies should be processed urgently.

**Table 1:**
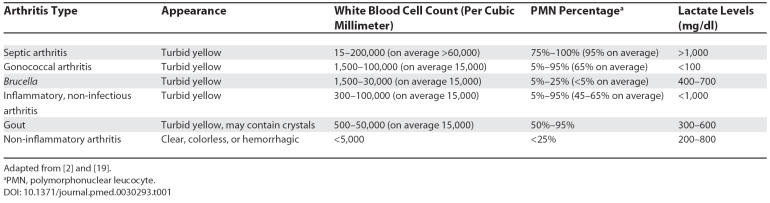
Synovial Fluid Characteristics in Arthritis

Blood cultures should be drawn to investigate an underlying bacterial infection or bacteremia. Given that brucellosis is a possible diagnosis, the blood cultures should be held for at least four to six weeks before a definite negative result is issued. Serology for typhoid, Lyme disease, and brucellosis should be performed, and a negative result, given the protracted period of disease, should have a high negative predictive value. Bone marrow cultures can be helpful in diagnosing brucellosis or typhoid. However, bone marrow biopsy is an invasive and painful procedure, and in this patient it should probably be withheld until a diagnostic dead end is reached.

An autoantibody profile should be performed, including rheumatoid factor, antinuclear antibodies, and anti-double-stranded DNA antibodies. Work-up for reactive arthritis, including serology for potential infectious triggers of the syndrome and even HLA B27 positivity, should be withheld until exclusion of other potential diagnoses. A serum ferritin value would be helpful: extremely high values are associated with adult-onset Still disease [3].

### Progress

**Figure 1:**
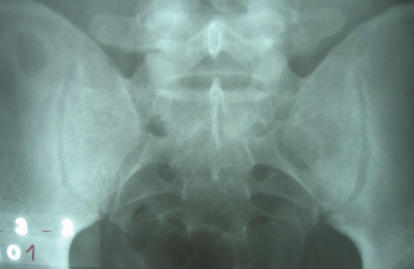
Plain Radiograph of the Sacroiliac Joints on Presentation to the Hospital San Juan de Matucana The radiologist reported this radiograph as normal.

A plain radiograph of the sacroiliac joints was reported as normal (Figure 1), as was a plain radiograph of the right sternoclavicular joint. There was no leucocytosis, but a relative lymphocytosis was present. A routine biochemistry profile was normal. Blood cultures were drawn. Serology for brucellosis was positive: the rose bengal test, a rapid screening test for *Brucella*, was positive, as were tube agglutination and 2-mercaptoethanol tests, in titers well above the diagnostic limits, thus further confirming the previous diagnosis of brucellosis.

### Shouldn't These Serological Test Results Be Expected, Given the Recent History?

It is true that serology alone cannot diagnose a relapse of brucellosis, since antibody titers tend to persist for a period of months to years. One would have to compare current titers with antibody titers obtained during resolution of the first episode, but such information was not available. However, serology was important in confirming that the original illness was indeed brucellosis.

### What Was the Likely Diagnosis at This Stage?

Although serology alone was not sufficient to make a diagnosis of brucellosis relapse, this diagnosis was supported by: (1) the clinical presentation—in particular, protracted fever and sacroiliitis, (2) the recent history of brucellosis, and (3) the absence of any strong indicators of an alternative diagnosis. Relative lymphocytosis in the absence of leucocytosis is also a common finding in brucellosis. Relapse of brucellosis should be further confirmed by blood cultures, or, more practically, by response to specific treatment.

**Figure 2:**
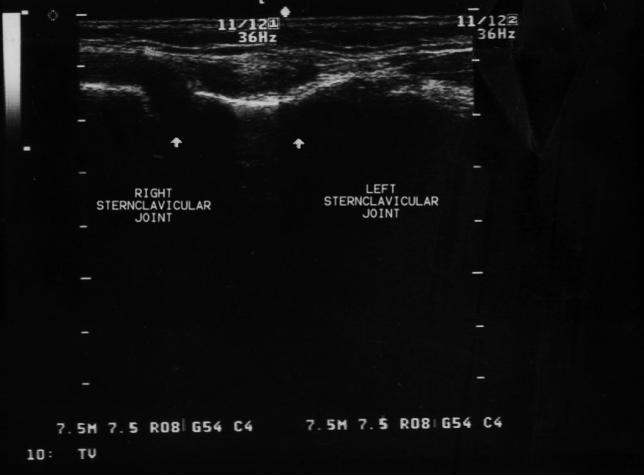
Ultrasonography of the Sternoclavicular Joints The image from an ultrasonography examination shows a normal left sternoclavicular joint, the clavicle appearing in continuity with the sternum. In the right sternoclavicular joint, the continuity is lost, reflecting the joint distention.

The patient was treated with streptomycin 1 g daily intramuscularly and doxycycline 200 mg daily. The result of the blood culture two weeks later showed a positive result for *Brucella melitensis*. Clinical response to treatment, in terms of fever and arthritis, was satisfactory. Three weeks later, at the end of streptomycin administration, he developed profound inflammation of the right sternoclavicular joint associated with pain and functional limitation. Ultrasonography showed significant distension of the right sternoclavicular joint capsule (Figure 2). An attempt to aspirate the right sternoclavicular joint was unsuccessful.

### What Is the Most Likely Final Diagnosis, and What Is the Treatment?

Had blood cultures been negative, the diagnosis of brucellosis would be seriously in doubt. With negative cultures, alternative diagnoses should once more be entertained, as discussed above, since the patient might suffer from brucellosis *and* another disease. In particular, a diagnosis of sternoclavicular septic arthritis should be considered, which is due to *Staphylococcus aureus* in half of all cases [4]. In one review of 180 cases of sternoclavicular septic arthritis, the mean age of patients was 45 years, three-quarters were male, and two-thirds were febrile [4]. Patients presented with chest pain (78%) and shoulder pain (24%) after a median duration of symptoms of 14 days. Risk factors included intravenous drug use (21%), distant site of infection (15%), diabetes mellitus (13%), trauma (12%), and infected central venous line (9%), although there was no obvious risk factor in 23% of the patients.

Treatment failure in brucellosis is the most likely diagnosis for this patient, and the antibiotic regimen should be modified or intensified. The patient had initially responded to a regimen of doxycycline and rifampin when he was first diagnosed with brucellosis, and a similar regimen should have been prescribed when he presented with a likely diagnosis of brucellosis relapse. Reactive arthritis remains a possible diagnosis in this patient, perhaps triggered by the initial infection with *Brucella* (rather than a gastrointestinal or genitourinary infection), although it is unclear whether reactive arthritis can be triggered by Brucella infection [5]. Streptomycin treatment was withheld, and he was given triple therapy with oral rifampin 600 mg four times daily, doxycycline, and ofloxacin 800 mg four times daily for eight weeks, according to local practices.

### How Should the Follow-Up of the Patient Be Scheduled?

A rapid response to antibiotic treatment is expected soon after the initiation of appropriate antibiotic treatment. Follow-up should ensure adherence to treatment duration. Proof of microbiological eradication, as demonstrated by negative blood cultures, is of little value in brucellosis, as is serology, as discussed. The patient should be advised to seek medical advice only in the event of symptom reappearance. In the present case, a three-year follow-up was uneventful; a gradual decrease in serology titers was noted on repeated testing.

## DISCUSSION

**Figure 3:**
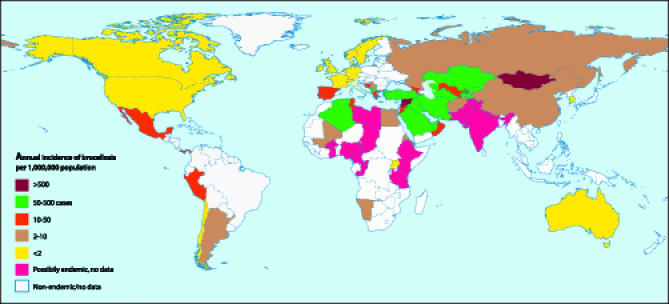
The Global Endemicity of Brucellosis Derived from [7].

Brucellosis is an anthropozoonosis caused by species of the genus *Brucella*, gram-negative bacteria possessing unique pathogenetic characteristics [6]. There has been a renewed interest in the disease recently, due to the change in its global distribution [7], the small but continuous influx of cases into the developed world through food importation and international travel [8], and the potential use of *Brucella* as a biological weapon. The disease is prevalent in the Near East and Central Asia, the Mediterranean, and certain Latin American countries such as Mexico and Peru (see Figure 3), the latter being the origin of the infection in this case report.

### 

#### Pathogenesis

Human disease is mainly attributed to *B. melitensis*, a pathogen of sheep, goats, and camels, transmitted to humans through consumption of unpasteurized dairy products, direct contact with infected animal tissues (characteristically the placenta), and generation of aerosols. The cattle pathogen *B. abortus* is nowadays less commonly associated with human disease.

#### Clinical features

Human brucellosis is characterized by a diversity of clinical presentations [9] and a predisposition to chronicity. This predisposition is attributed to the ability of the pathogen to reside in specialized compartments of phagocytes without interrupting their functioning, thus minimizing the interaction with the host's immune system [10].

The disease typically presents as a non-specific protracted febrile syndrome but can also present as every focal complication imaginable (Table 2). The commonest complications include osteoarticular involvement, hematological abnormalities [11], and epididymo-orchitis. Spondylitis, neurobrucellosis (a diverse group of syndromes), and endocarditis are the more serious complications.

**Table 2:**
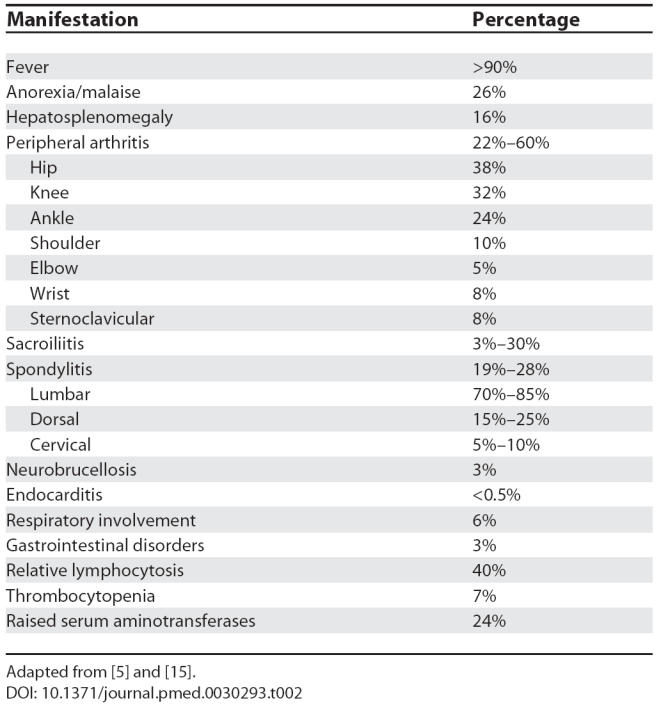
Clinical Manifestations of Brucellosis

Osteoarticular involvement can take the form of peripheral arthritis [12,13], sacroiliitis [14], or spondylitis [15]. The prevalence of osteoarticular involvement varies greatly in different case series, ranging from 2% to 85%, usually between 20% and 40%. Most cases manifest as monoarthritis of the large lower-extremity joints. Sternoclavicular arthritis due to *Brucella* is an unusual localization: the largest series presented in the literature consisted of ten patients [12], and less than 100 cases have been described in the literature in total, often as isolated case reports [16–18]. Diagnosing brucellosis as the cause of sternoclavicular arthritis is an easy task when in the context of an overall compatible clinical picture, yet arthritis may be the predominant syndrome, thus underlining the importance of including brucellosis in the differential diagnosis of practically any clinical syndrome in endemic areas.

#### Diagnosis

Diagnosis of brucellosis is made by isolation of the organism from blood (success ranging from 10% to 70%) and bone marrow [19], or joint fluid in cases of arthritis. As shown in Table 1, raised lactate levels in the joint aspirate suggest a septic arthritis (though raised lactate is not specific to brucellosis) [20].

Various serology tests allow for accurate diagnosis. These assays may detect: (1) antibodies against surface antigens; such assays include the serum agglutination test, and its variants the 2-mercaptoethanol test and the rose bengal test, or (2) antibodies against cytoplasmic proteins (the enzyme-linked immunosorbent assay) [21]. Both types of assay are sensitive and specific to ranges of 80% to more than 90%. However, these assays are inadequate for patient follow-up and in diagnosing relapses, due to difficulties in differentiating active versus past disease as a result of the extremely low decline in antibody titers through time. Widespread employment of newer techniques such as polymerase chain reaction assays [22] will allow for more accurate and rapid diagnosis in the future.

#### Treatment

The usual antibiotic combination therapy regimens given for brucellosis are doxycycline plus rifampin or doxycycline plus streptomycin [23,24]. Other alternative approaches use gentamicin, trimethoprim-sulfamethoxazole, and various quinolones (Box 2) [25]. Treatment duration matters, as shorter durations of therapy are associated with increased percentages of relapses [26]. 5%–15% of patients experience a relapse, usually during the first year of follow-up. Certain features of the initial infection make relapse more likely, including bacteremia, short disease duration, male sex, thrombocytopenia, and inappropriate treatment regimens [27].

**Box 2. Treatment Regimens for Brucellosis**

**Adults, uncomplicated**

Doxycycline 100 mg (twice daily) BID for six weeks and rifampin 600–1,200 mg daily for six weeks

*or*

Doxycycline 200 mg BID for six weeks and streptomycin 1 g daily intramuscularly for two to three weeks

**Alternative regimens**

Gentamicin 5 mg/kg daily for five to seven days, intravenously or intramuscularly, instead of streptomycin

*or*

Trimethoprim-sulfamethoxazole, 960 mg BID for six weeks, instead of doxycycline or rifampin

**Spondylitis/endocarditis/neurobrucellosis**

Protracted regimens (>12 weeks), triple or quadruple regimens, surgical intervention if needed

**Children <8 years**

Trimethoprim-sulfamethoxazole 18–24 mg/kg BID and rifampin 10–15 mg/kg daily, both for six weeks

**Pregnancy**

Rifampin 600–1,200 mg daily for six weeks as monotherapy, or in combination with trimethoprim-sulfamethoxazole 960 mg BID for six weeks

**Learning Points**

- Brucellosis is the commonest anthropozoonosis worldwide; it sometimes presents in the developed, non-endemic world.
- Think of brucellosis as a possible cause of arthritis, including sternoclavicular arthritis.
- Relapses are common in brucellosis, but do not represent treatment failure, and should be treated with the same regimens as the treatment of the initial disease.
- A variety of diagnostic tests exists, but patient follow-up is most accurate when performed clinically.
- In patients presenting with acute or sub-acute oligo- or pauci-arthritis a diagnostic joint aspiration is warranted, even in the presence of longstanding, pre-existing systemic arthritis.

#### Treatment of relapses

Eradication of brucellosis is difficult due to its residence in phagocytes, and the emergence of relapse does not necessarily equal treatment failure. The existing medical literature supports the use of the same regimens for treating relapse as for treating the initial infection, although anecdotal data from various centers support monotherapy (for example, a short doxycycline course in an uncomplicated relapse). In the present case report, alteration of the initial regimen led to a rapid second relapse. The subsequent regimens used reflected the individualized approach to brucellosis treatment by the various specialists worldwide, an approach that still leaves unanswered questions about the optimal treatment of human brucellosis [24].

## Abbreviation

BID: twice daily
